# Supplementary material for: Thermal Behavior and Smoke Suppression of Polyamide 6,6 Fabric Treated with ALD-ZnO and DOPO-Based Silane
Source: Materials (Basel). 2025 Jul 7;18(13):3195. doi: 10.3390/ma18133195 (PMC12250798; doi:10.3390/ma18133195)
Supplement: Supplementary file 1 [file materials-18-03195-s001.zip › materials-3658245-supplementary.pdf]

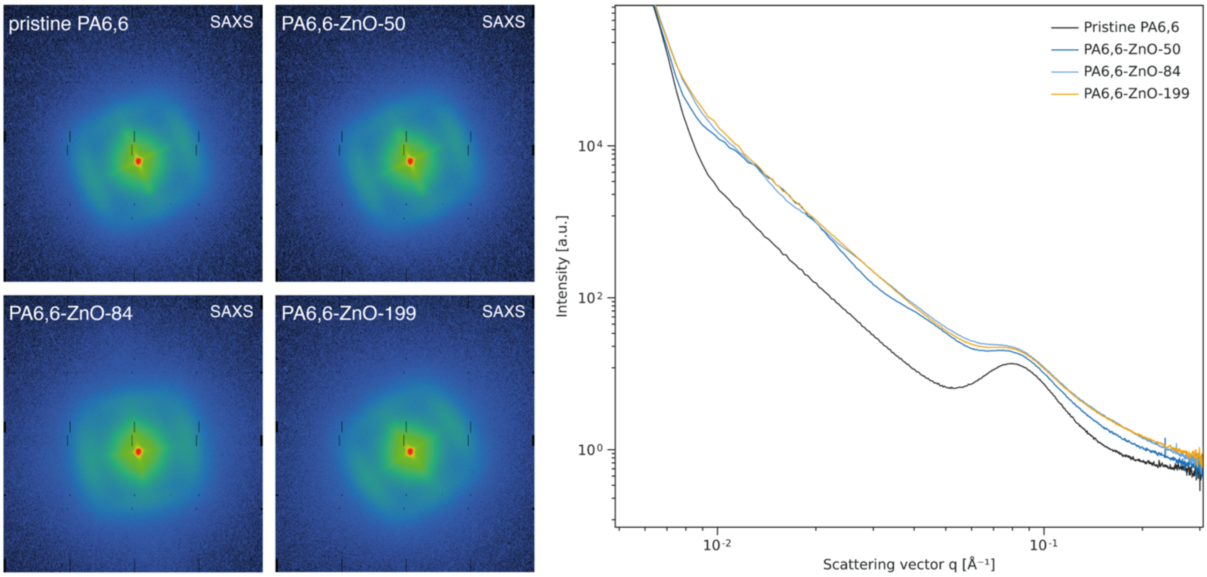

Figure S1. 2D small-angle X-ray scattering (SAXS) patterns of pristine and ZnO-coated PA6,6 fabrics (*left*), showing a systematic increase in scattering intensity with increasing ZnO layer thickness, indicating the formation of ZnO-based nanostructures. The corresponding 1D SAXS line profiles (*right*), derived from azimuthal integration, reveal that the broad peak centered around q ≈ 0.80 $Å^{-1}$ in pristine PA6,6 becomes less pronounced upon coating, suggesting modifications in local surface morphology and polymer interfacial ordering induced by the deposited inorganic layer.

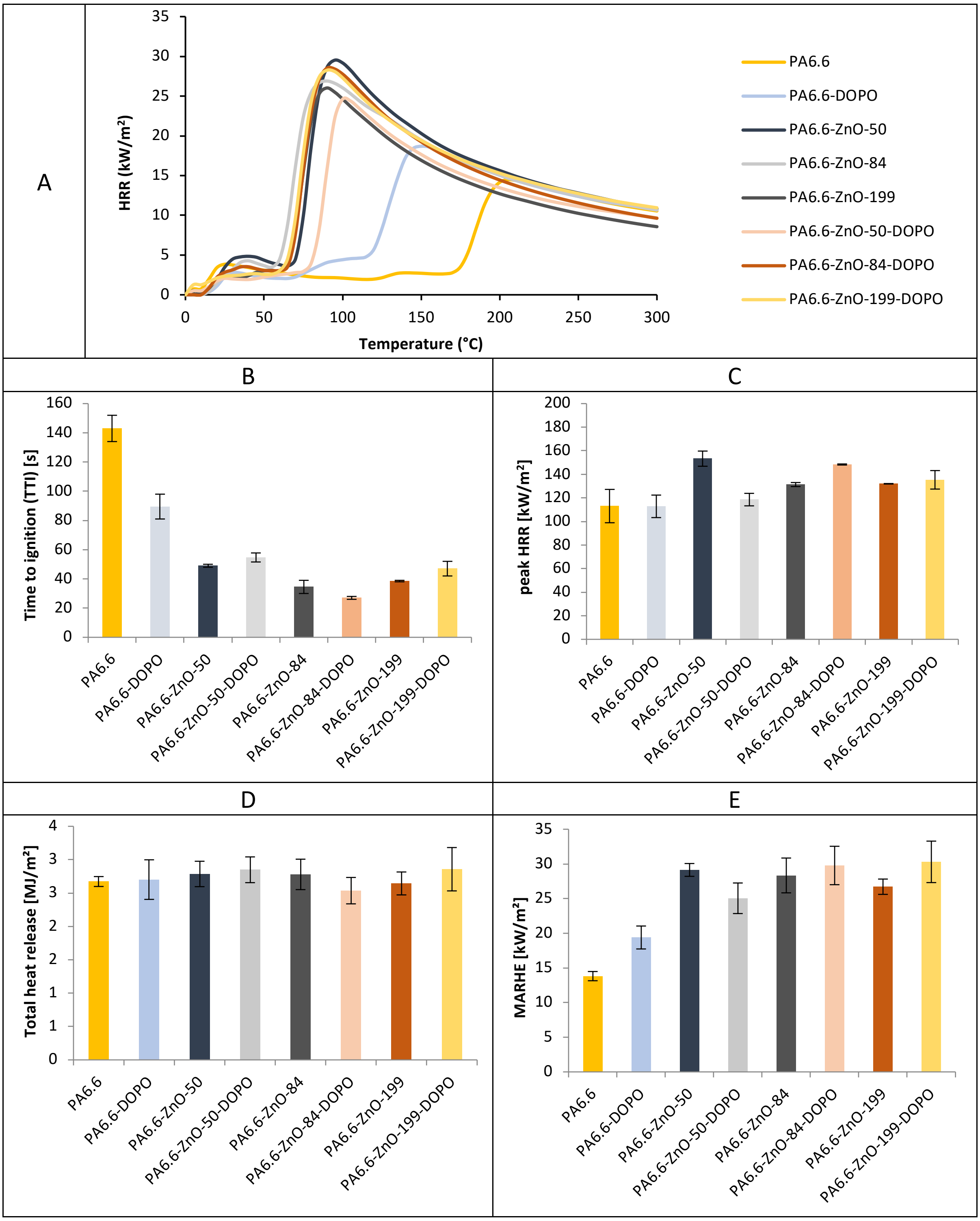

Figure S2. (A) Heat release rate (HRR) curves as a function of temperature, (B) time to ignition (TTI), (C) peak heat release rate (pHRR), (D) total heat released (THR), and (E) maximum average rate of heat emission (MARHE) of pristine PA6,6 and coated fabrics. Samples include ZnO-coated fabrics at varying layer thicknesses (50, 84, and 199 nm) and dual-layer coatings combining ZnO and DOPO-ETES. The measurements were conducted under a constant heat flux of 35 kW/m^2 using cone calorimetry.

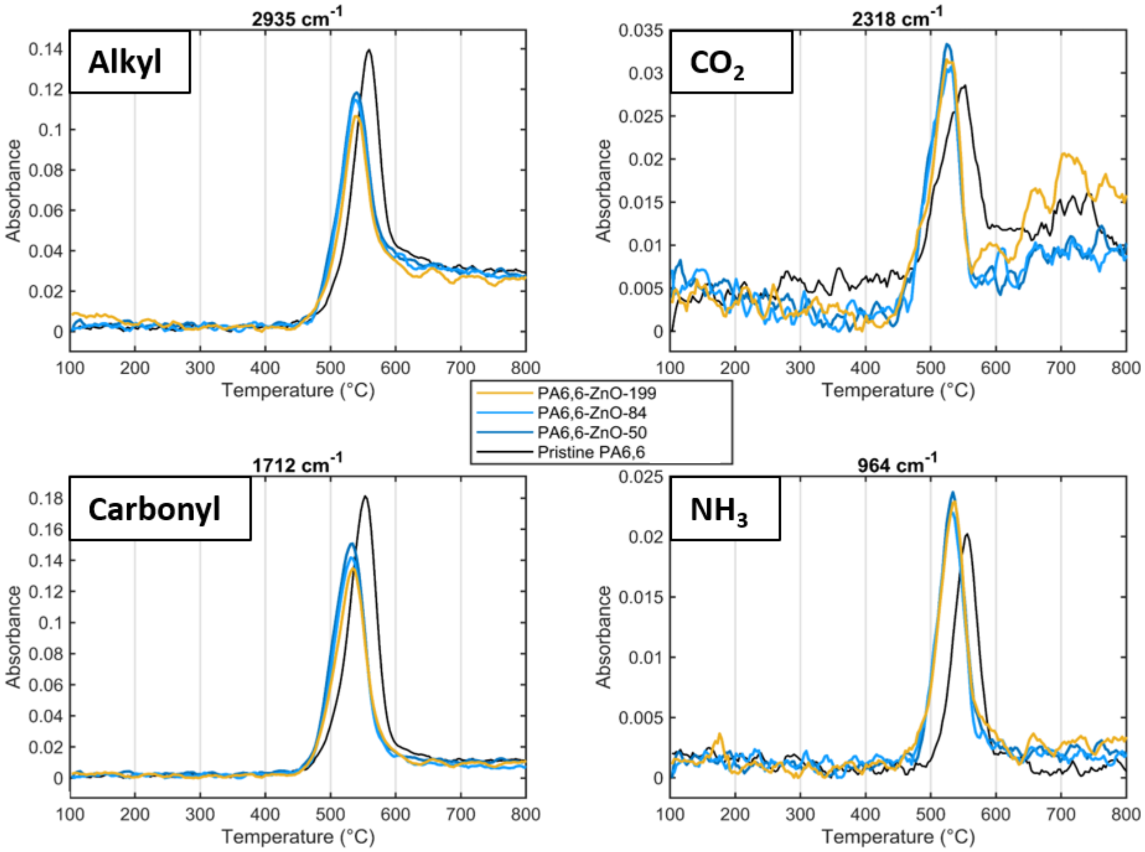

Figure S3. Temperature-resolved TG-IR absorbance profiles of key gaseous degradation products evolved from pristine and ZnO-coated PA6,6 fabrics. The presence of ZnO coatings reduce the emission intensity of alkyl and carbonyl species with increasing layer thickness, indicating suppressed release of combustible volatiles.

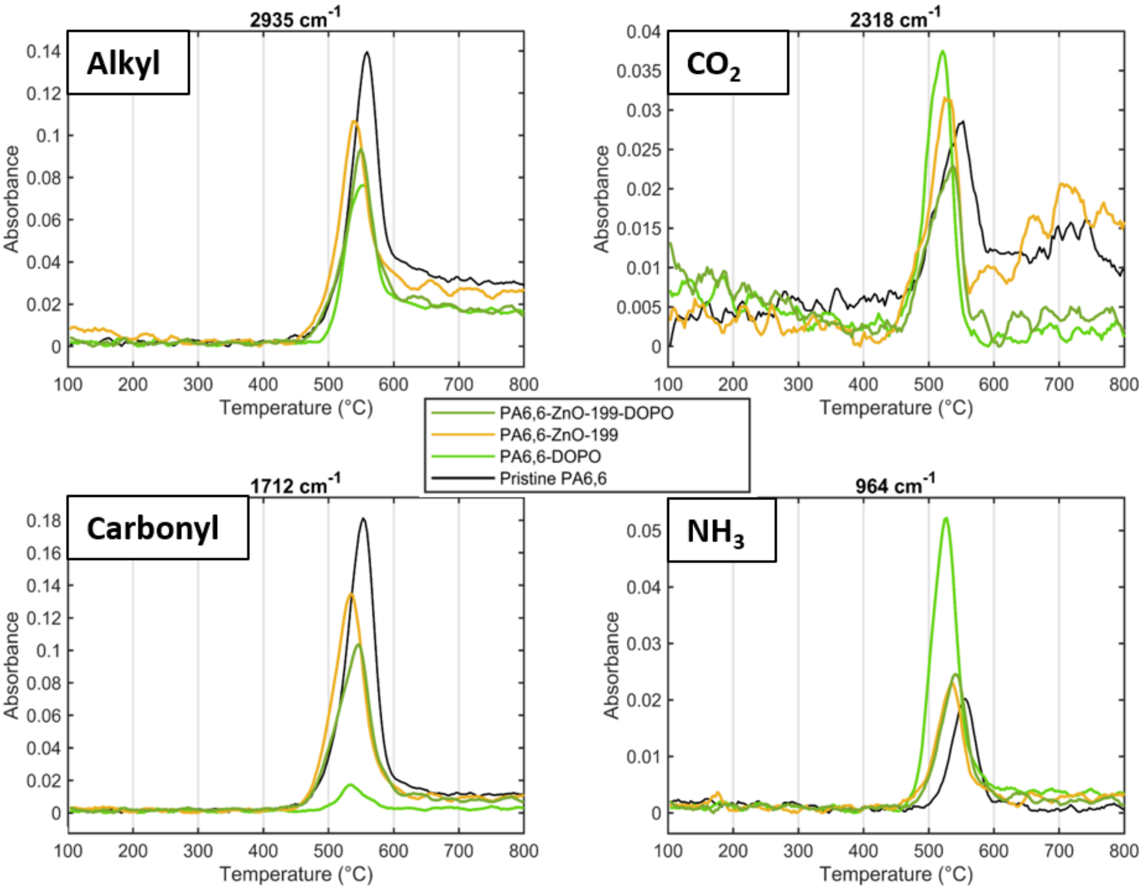

Figure S4. Temperature-resolved TG-IR absorbance profiles of volatile degradation products from pristine PA6,6, DOPO-treated fabric, ZnO-treated fabric with 199 nm thickness, and the combined ZnO-DOPO-treated fabric. The DOPO-treated fabric exhibits the strongest suppression of alkyl and carbonyl species compared to all other samples, including the pristine and ZnO-treated fabrics. While ZnO treatment alone leads to moderate volatile suppression, the combination of ZnO and DOPO does not further reduce alkyl or carbonyl emissions beyond the effect of DOPO alone, indicating no synergistic behavior. In terms of NH_3 release, the DOPO-treated fabric shows the highest emission. The ZnO-DOPO-treated fabric emits slightly more NH_3 than the ZnO-treated one, but significantly less than the DOPO-treated fabric.
